# Multi-Regional Natural Aging Behaviors and Degradation Mechanisms of Polyurethane-Coated Fabrics Under Coupled Multiple Environmental Factors

**DOI:** 10.3390/polym17192634

**Published:** 2025-09-29

**Authors:** Siying Wang, Dengxia Wang, Qi An, Jiakai Li, Kai Chong, Xinbo Wang, Jingjing Liu, Keyong Xie, Xuejun Hou, Jian Hou, Yan Sun

**Affiliations:** 1 Shandong Institute of Non-Metallic Materials, Tianzhuang East Road No. 3, Jinan 250031, China; 2 National Key Laboratory of Marine Corrosion and Protection, Luoyang Ship Material Research Institute, Wenhai Road, Qingdao 266237, China

**Keywords:** polyurethanes, coating, fabrics, natural aging, degradation, mechanism

## Abstract

Polyurethane-coated fabrics are widely employed as tarpaulin materials. However, due to the long duration and large space requirements of natural exposure tests, studies on fabric degradation remain scarce. To systematically investigate the natural aging patterns and mechanisms of polyurethane-coated fabrics, this study conducted 24-month natural aging tests in three representative regions: Xishuangbanna (tropical monsoon climate), Xiamen (subtropical maritime monsoon climate), and Jinan (temperate monsoon climate). Changes in appearance, mechanical properties, surface morphology, elemental composition, and microstructure were thoroughly analyzed. The results indicated that gloss decreased by over 60%, the color difference exceeded 5.8, and tear strength was reduced by more than 50%. SEM, ATR-FTIR, and XPS analyses revealed that hydrolysis and oxidation occurred in the coating, leading to coating thinning, fiber exposure, and even damage. In Xishuangbanna, high temperature, high humidity, and strong solar radiation are responsible for the most severe degradation of fabrics. High temperature, humidity, and salt fog synergistically accelerated the aging process. In Jinan, significant thermal strain contributed to deterioration, and fabrics exhibited the mildest degradation. This multi-region natural exposure study realistically simulates in-service aging behavior, providing important validation for accelerated laboratory aging methods, product reliability improvement, and service-life modeling.

## 1. Introduction

Covering equipment surfaces with fabrics is a commonly used protection method due to its simple process, low cost, easy application, independence from substrate geometry, and suitability for mass production [1,2]. It has been widely adopted for ground equipment [3]. Polyurethane contains chemical structures such as ether bonds, carbamate groups, ester groups, and benzene rings [4,5,6]. As a result, coatings made from it exhibit excellent adhesion, mechanical properties, abrasion resistance, corrosion resistance, chemical resistance, and gloss retention, occupying a critical role in fields including national defense, construction, marine, and aerospace industries [7,8]. During long-term service, these fabrics are exposed to harsh conditions such as high temperature, sunlight, seawater, rain, snow, and windblown sand. However, as polymeric compounds, both the fabric coatings and the substrate are inevitably subject to aging. The coupling of multiple factors in natural environments leads to molecular chain relaxation, migration of fillers or additives, and chain scission or cross-linking, resulting in aging behaviors such as loss of gloss, discoloration, embrittlement, coating peeling, and decline in mechanical properties. These degradation phenomena ultimately affect product quality and equipment safety [9,10,11]. Therefore, conducting environmental tests is of great significance for product development, accurate service-life prediction, full exploitation of material potential, and avoidance of major operational risks.

Research methods for studying material aging primarily include laboratory accelerated aging tests and natural exposure tests. Laboratory accelerated aging tests aim to simulate actual service conditions in the lab and accelerate the material aging process by intensifying specific factors [12]. Widely studied laboratory accelerated aging methods include thermal-oxidative aging, fluid resistance aging, radiation aging, and biodegradation [13,14,15]. Lu Pengcheng et al. [16] conducted hygrothermal aging, UV radiation aging, and oil resistance aging tests on polyurethane elastomers and observed that the elastomer was highly susceptible to UV radiation and oxidation. Eun Yeop Choi et al. [17] performed simulated seawater aging tests on thermoplastic polyurethane sealants and predicted their service life. Laboratory accelerated aging tests offer advantages such as short duration, high efficiency, and low cost. Nevertheless, they have difficulty replicating the coupled effects of complex factors encountered during actual service. This limits the ability to accurately reflect natural aging mechanisms and correctly predict natural aging behavior [18,19,20]. Natural exposure tests involve exposing materials directly to natural environments like the atmosphere, seawater, or soil to assess aging. Yunting Li et al. [21] performed natural aging tests on polyvinylidene fluoride (PVDF)-coated fabrics. The findings indicated that on the sun-exposed surfaces, the coating exhibited cracking, blistering, and chalking. On the shaded surfaces, blistering was observed, and the overall fabric brightness was reduced. Tear strength, Young’s modulus, and adhesive strength were also decreased. Yang Hui [22] conducted a one-year natural environmental exposure test of thermoplastic polyurethane (TPU) in Xiamen. It was found that the UV aging test effectively simulated the aging behavior of TPU under natural conditions, with 5 h of artificial thermal-oxidative aging at 190 °C being approximately equivalent to 12 months of natural aging. However, due to the extended duration required for natural aging, no further data were obtained. Li Qianqian et al. [23] carried out a two-year natural test of polyurethane coatings in a coastal atmosphere. The gloss loss rate of the coating reached 68%, the color difference exceeded three, and the surface gradually became rougher. Natural aging tests are time-consuming. Additionally, regional climatic variations necessitate large test sites and significant investments in manpower and material resources. Nevertheless, they can more realistically reflect the aging process of materials in natural environments. Natural aging provides a highly valuable reference for understanding the aging process of components working or stored in natural environments [24]. Crucially, it offers essential data for establishing and validating laboratory accelerated aging test methods and service-life prediction models [22,23].

Current research has focused more on the biodegradation of polyurethane [25], the degradation of polyurethane elastomers [20], and the development of polyurethane coatings [26], while long-term natural aging tests on polyurethane-coated fabrics have been less frequently conducted. However, conducting systematic aging experiments on these fabrics, thoroughly investigating aging behaviors, and elucidating degradation mechanisms are of crucial importance for multiple stages including material development, product processing, service-life prediction, and safety evaluation. Consequently, we conducted 24-month natural aging tests on polyurethane-coated fabric samples in Xishuangbanna, Xiamen, and Jinan, analyzing changes in appearance, mechanical properties, microstructure, and surface morphology to investigate aging mechanisms and accelerating factors.

## 2. Materials and Methods

### 2.1. Sample Preparation

**Figure 1:**
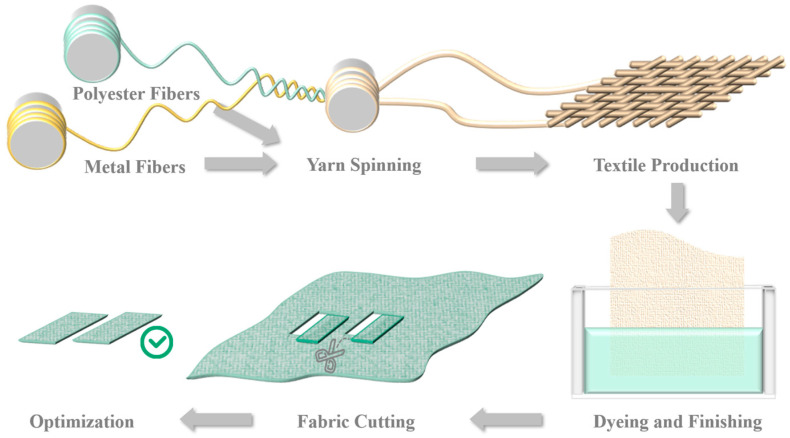
Fabric production process.

**Figure 2:**
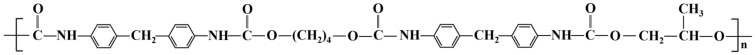
The repeat structure of the polyurethane.

As shown in Figure 1, polyester fibers and stainless-steel metallic fibers were blended and processed through carding and drawing to form yarn. The yarn underwent sizing and warping to produce plain-weave textiles. The textiles were from the supplier Taizhou Huarun Textile Co., Ltd. (Taizhou, China). This fabric was then dyed at high temperature and pressure, followed by the application of a polyurethane coating solution to its surface, producing a polyurethane-coated fabric. The coating was from China North Industries Group Corporation Limited (NORINCO) Institute 53 (Jinan, China). The repeat structure of the polyurethane is shown in Figure 2.

### 2.2. Natural Aging

The fabrics were subjected to natural environment exposure testing for 24 months, with sampling conducted at 0, 3, 6, 9, 12, 18, and 24 months. Exposure sites were located at Xishuangbanna (hereafter abbreviated as BN), Xiamen (abbreviated as XM), and Jinan (abbreviated as JN) in China. Site BN features a tropical monsoon climate characterized by abundant sunshine, low wind speed, mild winters, ample rainfall, and high temperatures. Site XM is characterized by a subtropical maritime monsoon climate featuring cool summers, mild winters, warm conditions, frequent rainfall, and persistently windy conditions year-round. Site JN exhibits a temperate monsoon climate with four distinct seasons: cold winters, hot summers, and unevenly precipitation distribution. Table 1 provides detailed climatic data.

### 2.3. Characterization

#### 2.3.1. Gloss

Specular gloss at 60° was measured according to GB/T 9754-2007 [27] using a gloss meter (3nh HG268 from 3nh Co., Ltd., Guangzhou, China). Six surface points per sample were tested. Five samples per site were measured, and average values were calculated.

#### 2.3.2. Color Measurement

Color was measured per GB/T 11186-1989 [28] using a spectrophotometer (3nh YS3020 from 3nh Co., Ltd., Guangzhou, China). Three sets of L*, a*, b* coordinates were recorded per sample. Color difference (ΔE*) was calculated using the CIELAB formula. Five samples per site were tested, and average ΔE was determined. The CIELAB color difference formula is as follows:

(1) $$\Delta E_{ab}^{*}={\left[{\left(\Delta L^{*}\right)}^{2}+{\left(\Delta a^{*}\right)}^{2}+{\left(\Delta b^{*}\right)}^{2}\right]}^{\frac{1}{2}},$$

where $\Delta L^{*}={L_{T}}^{*}-{L_{R}}^{*}$, $\Delta a^{*}={a_{T}}^{*}-{a_{R}}^{*}$, $\Delta b^{*}={b_{T}}^{*}-{b_{R}}^{*}$. ${L_{T}}^{*}$, ${a_{T}}^{*}$, ${b_{T}}^{*}$ are the chromaticity coordinates of the sample and ${L_{R}}^{*}$, ${a_{R}}^{*}$, ${b_{R}}^{*}$ are the color coordinates of the reference sample.

#### 2.3.3. Mechanical Properties

As fabrics frequently undergo tearing under concentrated loads during service, tear strength better reflects fabric durability. Therefore, tear resistance was tested per GB/T 3917-2009 [29] using trapezoidal samples on an electronic universal testing machine (Instron 5696 from Instron in Norwood, Massachusetts, USA). Tests were conducted with a 25 mm gauge length and 100 mm/min crosshead speed. Five samples per site were tested, and averages were calculated.

#### 2.3.4. Microstructure

Attenuated total reflection Fourier-transform infrared (ATR-FTIR) spectra of fabrics were acquired using a Fourier-transform infrared spectrometer (Thermo Scientific Nicolet iS50 from Thermo Fisher Scientific in Waltham, Massachusetts, USA). Scans were performed over the 4000–500 cm^−1^ range with 8 accumulations.

X-ray photoelectron spectroscopy (XPS) analysis of fabric surface components was performed using an X-ray photoelectron spectrometer (Thermo Fisher ESCALAB Xi+ from Thermo Fisher Scientific in Waltham, Massachusetts, USA). Monochromatic Al-Kα radiation (1486.6 eV) served as the excitation source at 0.1 eV step size, 15 kV tube voltage, and 10 mA beam current.

#### 2.3.5. Surface Morphology

Field-emission scanning electron microscopy (ZEISS Gemini SEM 300 from Carl Zeiss AG in Oberkochen, Germany) was employed. Samples were sectioned, mounted on aluminum stubs, and sputter-coated with gold. Surface and cross-sectional imaging utilized secondary electron and backscattered electron detectors. Magnifications were 100× and 1000× for secondary electron images (SEI) and 200× for backscattered electron images (BSE).

## 3. Results and Discussion

### 3.1. Appearances

**Figure 3:**
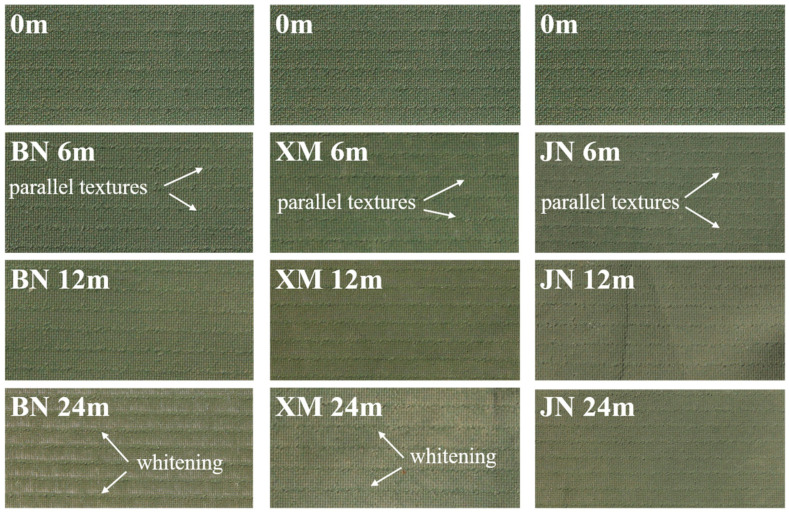
Macroscopic photos of the appearance of natural aging fabrics in different regions.

As shown in Figure 3, the original fabric exhibited a smooth surface with slight granular texture and uniform coating distribution. These photos were taken with a Canon EOS 500D in Jinan, China, at 10:00 a.m. Specific settings were as follows: 85 mm, F10, 1/400 s, ISO 100. During initial aging, distinct parallel textures emerged with increased roughness and color changes, presumably attributable to volatile substance release, which altered surface structures [14]. As the experiment progressed, fabric surface smoothness increased, while textures were more obvious. In later stages, significant color fading with yellowing, whitening, and overall dilution occurred.

In BN, fabric exhibited minimal changes during the initial stage. However, synergistic effects of high temperature and intense irradiation caused pronounced coating deterioration after 6 months, indicating the most severe aging among all sites. In XM, under high-humidity and high-salinity fog conditions, fabrics demonstrated the most significant initial changes. Subsequent aging stages remained relatively uniform. In JN, characterized by comparatively lower temperatures, weaker irradiation, and less precipitation, fabrics exhibited the mildest aging-induced alterations. Post-aging coatings retained greater integrity and smoothness.

### 3.2. Gloss

**Figure 4:**
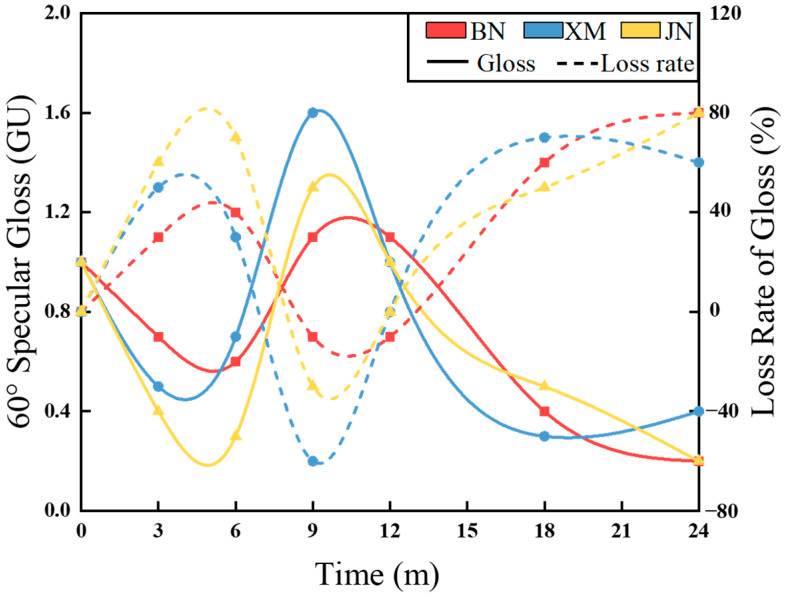
The curves of the fabrics’ 60° specular gloss during natural aging.

Figure 4 illustrates 60° specular gloss variation of fabrics during natural aging across three sites. Gloss displayed an initial decrease, followed by an increase and eventual reduction, with peak values consistently occurring at 9 months. This parameter is governed principally by intrinsic fiber luster, fabric architecture, and finishing methodologies [30]. In the early stage of aging, enhanced surface flatness due to rainfall impact and particle abrasion, combined with progressive resin coating curing, reduced diffuse reflection and increased gloss. Prolonged exposure caused coating degradation, thickness reduction, and roughness escalation, ultimately leading to fiber exposure and gloss attenuation [11,31]. After 24 months, the gloss retention rates were 20%, 40%, and 20% for BN, XM, and JN, respectively.

### 3.3. Color Measurement

**Figure 5:**
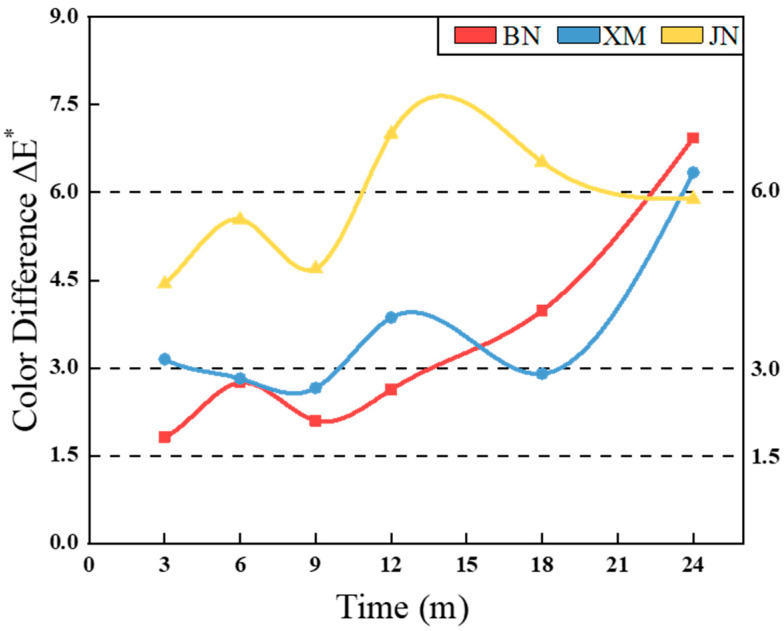
The curves of the fabrics’ color difference during natural aging.

As shown in Figure 5, fabric color difference (ΔE*) demonstrated a nonlinear upward trend with prolonged natural aging. Oxidation of polyurethane coatings generated new chromophores and induced yellowing [20,32]. Fluctuations in temperature and humidity affected the degradation kinetics, while varying degrees of chalking and thinning altered the surface morphology and caused optical inhomogeneities [33].

Divergent environmental conditions resulted in site-dependent aging mechanisms and severities. According to GB/T 1766-2008 Paints and varnishes—Rating schemes of degradation of coats, as detailed in Table 2, after 24 months of aging, fabrics exposed to the most aggressive conditions in BN exhibited the maximum ΔE* of 6.93, corresponding to distinct color change [34]. Fabrics in XM demonstrated moderate ΔE* at 6.34, similarly graded as distinct color change. The fabrics in the JN region had the least color difference (5.89), rated as a slight color difference.

### 3.4. Mechanical Properties

**Figure 6:**
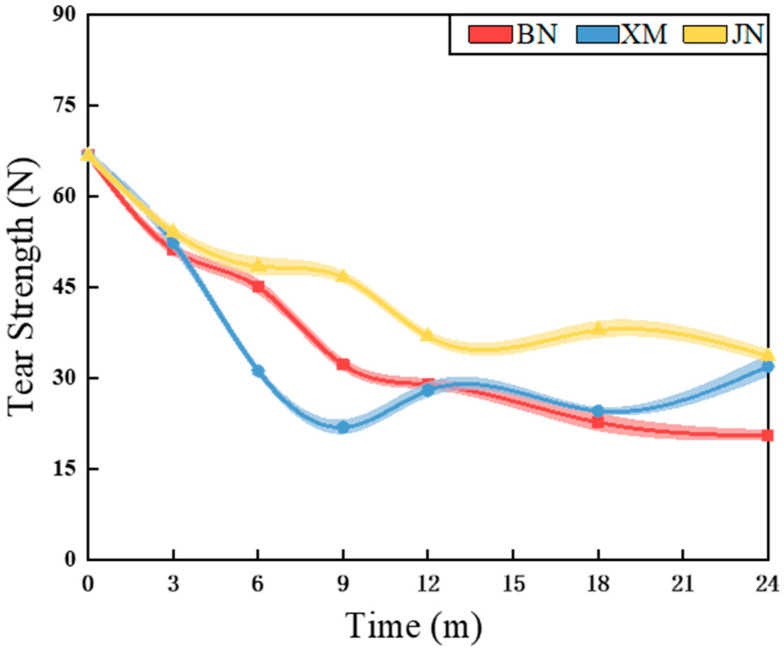
The curves of the fabrics’ tear strength during natural aging.

**Figure 7:**
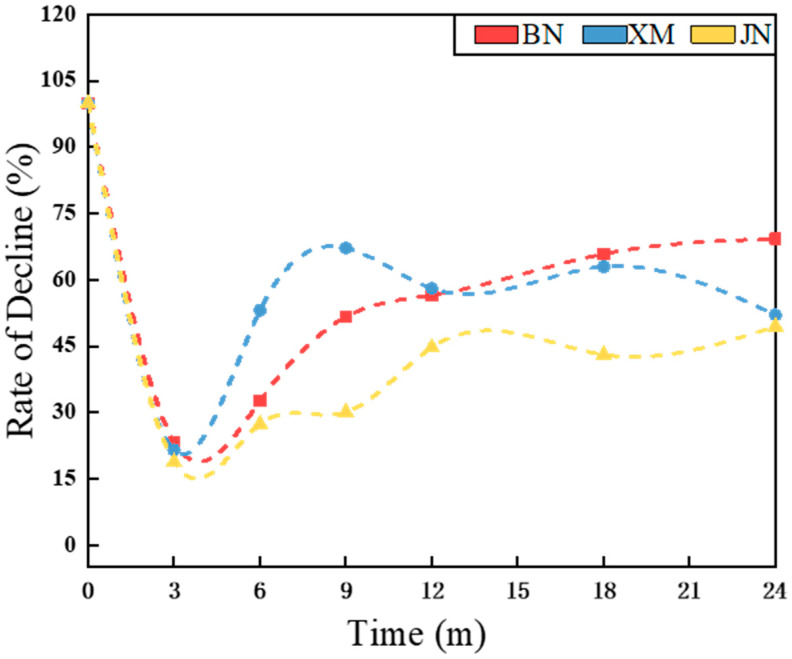
The curves of the fabrics’ tear strength decline rate during natural aging.

The tear strength profile of fabrics during natural aging is shown in Figure 6 and Figure 7. Fabric mechanical properties demonstrated an overall declining trend with natural aging duration, characterized by an initial rapid decline followed by a gradual decrease. In BN and XM, characterized by higher temperatures and stronger irradiation, tear strength decreased substantially within 9 months, while the fabric in JN exhibited accelerated reduction within 12 months. Coating technology served as a key enhancer of fabric mechanical performance [35]. During the early aging stages, coating oxidation and hydrolysis induced thinning and delamination, thereby causing rapid deterioration. As aging time increased, the subsequent deceleration of tear strength loss potentially stemmed from the temperature fluctuations and moisture ingress in natural environments, which promote molecular chain mobility and facilitate polymer chain reorganization and crosslinking [36]. Thus, this process alleviates internal stresses and defects and simultaneously enhances coating rigidity and improves fiber stress distribution uniformity [37]. Mechanical property retention rates reached 48% in BN after 9-month aging, 47% in XM following 6 months, and 50% in JN after 24-month exposure.

Notably, fabric degradation under natural conditions involves competing mechanisms, including beneficial effects such as post-curing and detrimental effects like hydrolysis [38,39]. The continued post-curing of polyurethane under suitable environmental conditions positively influences mechanical properties [40,41]. Moisture diffusion follows Fickian behavior [42], while the penetration of both oxygen and radiation becomes increasingly limited with depth, demonstrating the nonlinear nature of environmental aging factors [43,44]. The outer coating gradually peels away, exposing the underlying layer. Furthermore, corrosive ions react with metallic components in the fabric [45,46,47]. These interconnected processes collectively lead to fluctuations in mechanical performance.

### 3.5. Scanning Electron Microscopy (SEM)

**Figure 8:**
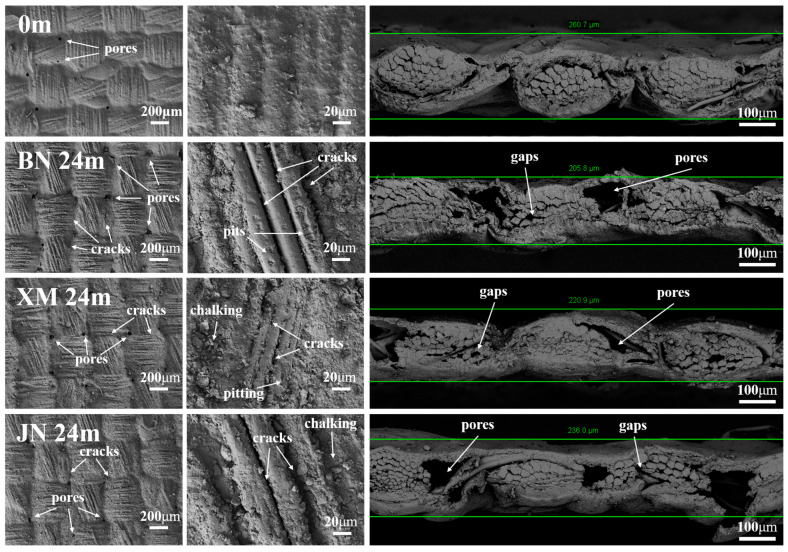
Fabric surface morphology.

Figure 8 presents SEM images of the fabrics. The original fabric exhibits a smooth, dense surface. The coating is uniformly applied without any cracks or delamination. There are a few pores, which are thought to be because of volatile substances escaping during processing. Fibers are tightly aligned and fully encapsulated by the coating without exposure. The original fabric thickness was 260.7 µm. After 24 months of natural aging, fabrics in BN displayed significantly increased surface roughness. Fine cracks and multiscale pores developed at fiber bundle intersections. Extensive fiber exposure occurred, accompanied by fiber surface pitting and inter-fiber gaps. The fabric thickness was critically reduced to 205.8 µm. Surface flatness of samples in XM markedly decreased. Severe coating chalking was observed. Though residual coating fragments remained, small pits appeared at fiber bundle junctions with partial fiber exposure. Fiber arrangement became loosened with abundant gaps. The fabric thinned noticeably to 220.9 µm. Samples in JN displayed the mildest surface roughness increase. Coatings exhibited cracking and chalking. Sparse micro-pores formed at fiber bundle intersections. Inter-fiber gaps emerged while partial coating coverage persisted. Coating thinning was least pronounced, with a fabric thickness of 236.0 µm.

Coating oxidation, hydrolysis, chalking, and delamination collectively drive alterations in gloss and color difference. These expanding defects also facilitate oxygen and water, which further accelerates fabric degradation [13]. Concurrently, coating thinning, fiber structural damage, and weakened coating–fiber interfacial adhesion synergistically compromise mechanical properties. SEM analyses collectively indicate the most severe aging in BN, intermediate degradation in XM, and relatively minor deterioration in JN. The order of degradation severity is consistent with the differences in appearance changes reduction as mentioned above.

### 3.6. Attenuated Total Reflection Fourier-Transform Infrared Spectroscopy (ATR-FTIR)

**Figure 9:**
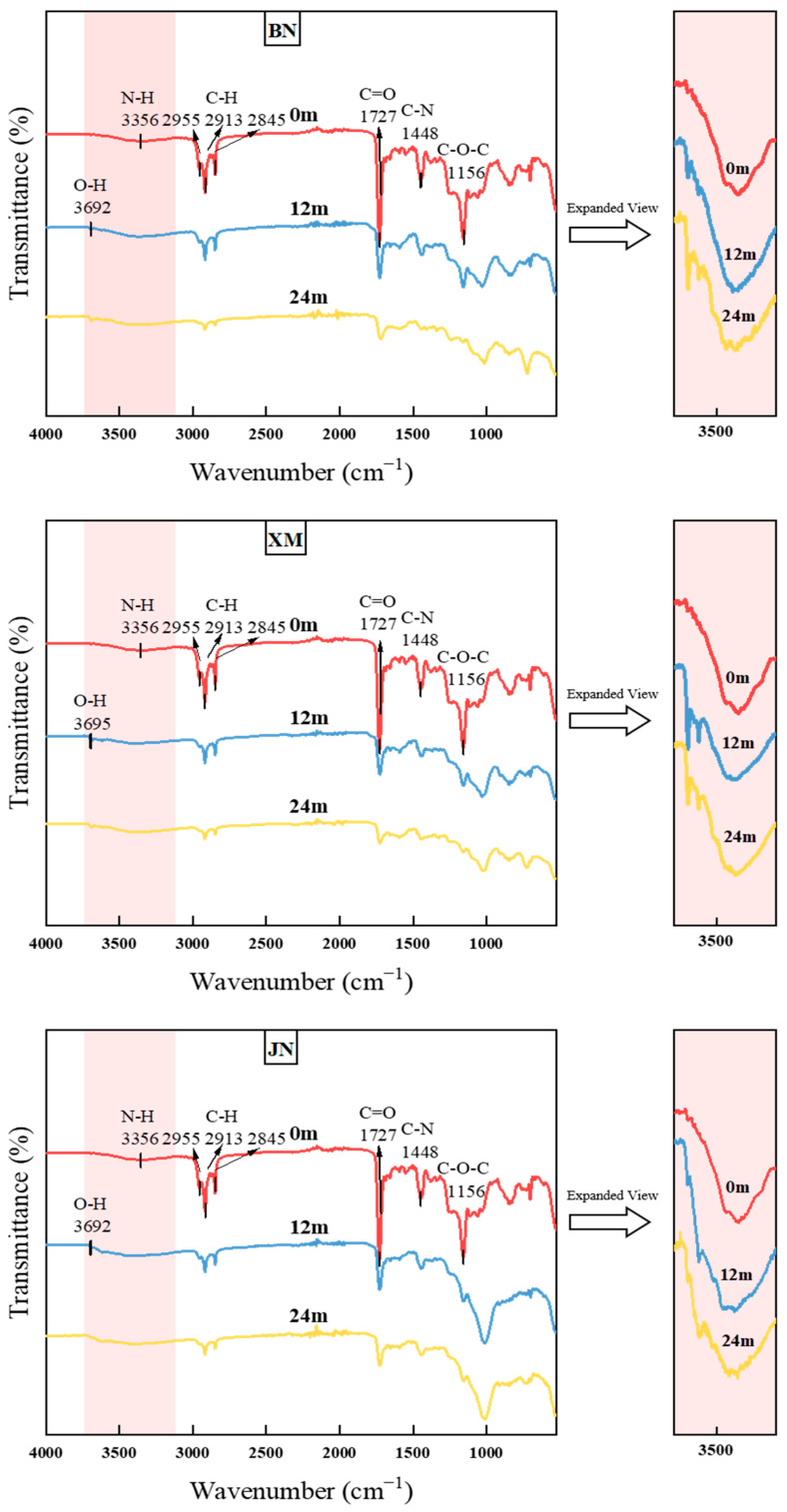
ATR-FTIR spectrums of fabrics during natural aging.

As shown in Figure 9, the transmittance peak at 3356 cm^−1^ is assigned to N–H stretching vibration. Peaks at 2955, 2913, and 2845 cm^−1^ correspond to C–H stretching vibrations. The 1727 cm^−1^ peak represents C=O stretching vibration, while 1687, 1656, and 1595 cm^−1^ are attributed to aromatic ring skeletal vibrations. Transmittance at 1552 cm^−1^ indicates N–H bending vibration in urethane groups. The 1448 cm^−1^ peak arises from C–N stretching vibrations, 1379 cm^−1^ from C–H bending of methyl groups (–CH_3_), and 1156 cm^−1^ to C–O–C stretching vibration.

After natural aging, a new peak emerges at 3692 cm^−1^, assigned to O–H stretching vibration [14]. The transmittance at 1727 cm^−1^ increases significantly, likely due to C=O oxidation or urethane hydrolysis, which reduced carbonyl groups and generating hydroxyls [48,49,50]. The N–H stretching vibration at 3356 cm^−1^ demonstrates decreased transmittance, potentially originating from –NHCOO– hydrolysis forming amino groups (–NH_2_). Increased transmittance at 1156 and 1011 cm^−1^ suggests oxidative/hydrolytic cleavage of C–O–C bonds weakening stretching vibrations [16,51]. Elevated transmittance at 2957, 2915, and 2849 cm^−1^ indicates alkyl chain scission reducing C–H group density [11]. Furthermore, the coupled effects of irradiation, water, and oxygen during prolonged natural exposure thin coatings and decrease functional group concentrations, universally reducing infrared absorption transmittance and increasing transmittance intensity [52].

With progressive aging in BN, transmittance of C=O at 1727 cm^−1^ increased to 83.08%, which indicated the most severe carbonyl degradation. Concurrently, the 1156 cm^−1^ transmittance rose to 81.63%, suggesting C-O-C accelerated cleavage. This likely attributed to synergistic oxidation and hydrolysis under high-temperature and intense irradiation. C=O transmittance of samples in XM reached 85.53% at 1726 cm^−1^, reflecting C=O observable degradation. The transmittance of the C–O–C peak at 1156 cm^−1^ increased to 81.15%, confirming the bond cleavage. Due to the coupled effects of high humidity, precipitation, and salt spray, intense hydrolysis of –NHCOO– and C–O–C bonds occurred. The transmittance of samples from JN increased to 79.16% at 1727 cm^−1^ and to 71.50% at 1156 cm^−1^, which shows a relatively small increase, pointing to a less severe degree of degradation. Notably, hydrogen bond peaks near 3618 cm^−1^ intensified in both BN and XM, reflecting enhanced hydrogen bonding during hydrolysis.

### 3.7. X-Ray Photoelectron Spectroscopy (XPS)

**Figure 10:**
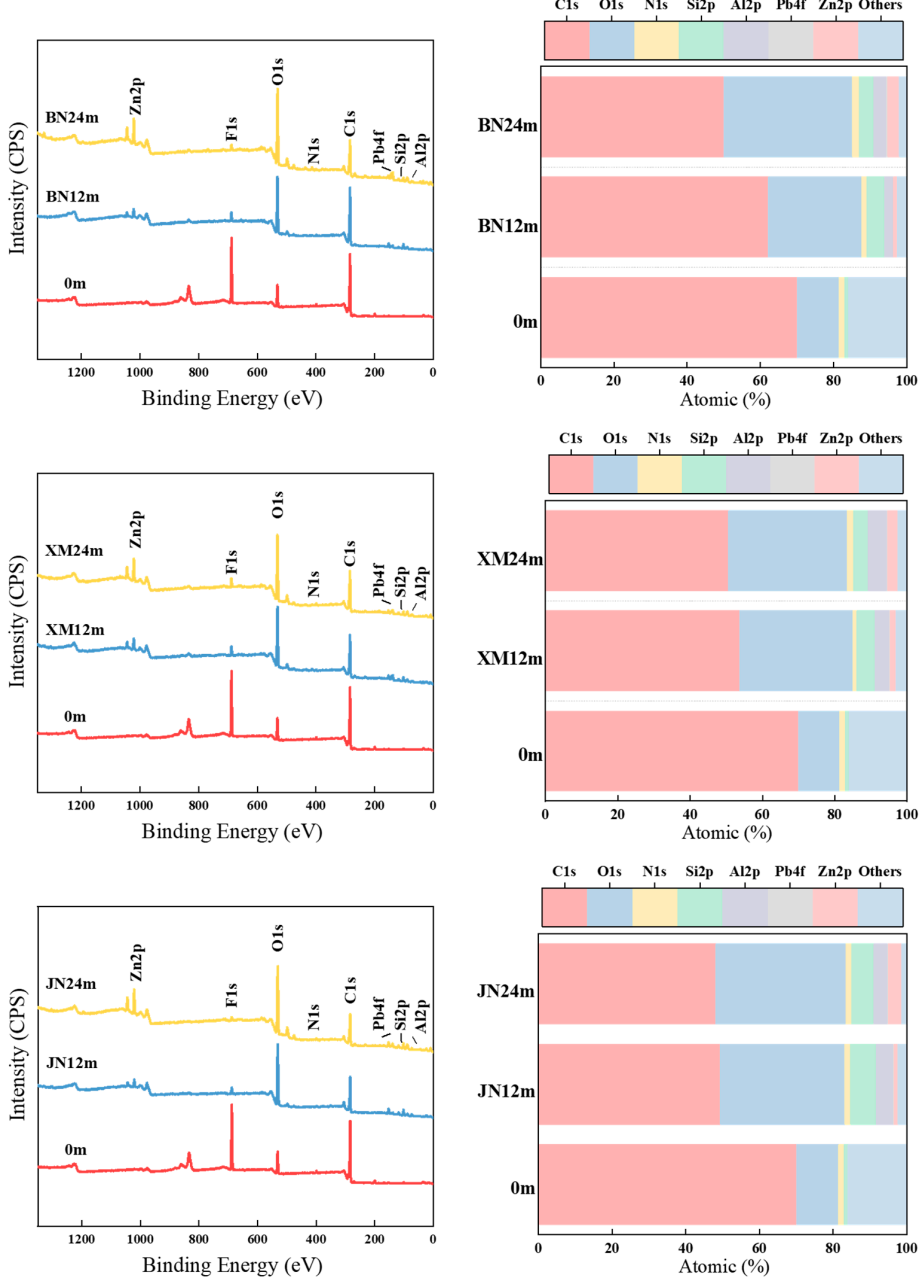
XPS spectrums and diagrams of element relative contents of fabrics during natural aging.

Figure 10 presents XPS spectra and elemental changes of naturally aged fabrics. After natural aging, the C1s peak intensity decreased, accompanied by a slight leftward shift in binding energy. The O1s peak intensity increased. The Al2p, Zn2p, and Pb4f characteristic peaks appeared, and their intensities continued to increase with aging. Meanwhile, carbon content markedly decreased, whereas oxygen content substantially increased. These findings indicate that oxidation of C–C/C–H bonds reduces electron density around these bonds, leading to higher binding energies. As a result, the coating oxidation continuously deepened. Moisture ingress caused coating delamination, decomposition, shedding, and fiber exposure. In addition, metal elements like Al, Zn, and Pb from pigments gradually emerged, and their content correlated positively with aging time [45,53,54].

The BN region maintains relatively stable temperatures year-round, characterized by high temperatures, high humidity, and intense solar radiation. Under these conditions, oxidation and hydrolysis reactions occur continuously, leading to a steady decrease in carbon content and a gradual increase in oxygen content. In XM, the high concentration of salt spray in the atmosphere facilitates Cl^−^ and other ions entering and inducing electrochemical corrosion, accelerating the detachment of the coating from the substrate, leading to the exposure and accumulation of metallic elements [55]. Consequently, aging damage progressed rapidly, severely, and extensively. During later stages of aging, the reduction in oxidation and hydrolysis reactions, coupled with the deposition of metallic oxides onto the fabric surface, partially inhibiting further aging. This decelerated the rate of carbon loss and oxygen gain. JN experiences significant thermal stress due to temperature fluctuations promoting coating cracking [13]. Pathways for water and oxygen ingress were created, thereby accelerating the initial aging rate and causing dramatic early-stage fluctuations in carbon and oxygen content. Furthermore, the inherently higher silicon content in this region, combined with increased binding energy post-aging, led to the formation of silicon oxides. These oxides exert a barrier effect on the fabric, mitigating the aging rate in later stages.

### 3.8. Degradation Mechanisms

**Figure 11:**
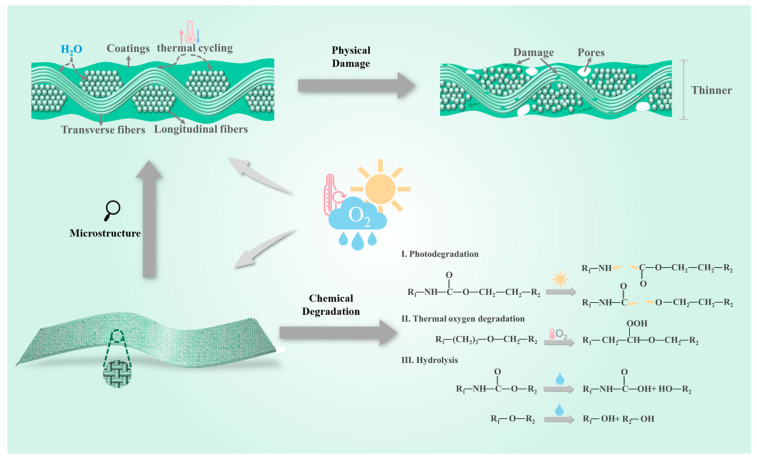
Coating degradation mechanism.

Figure 11 illustrates aging mechanisms of polyurethane-coated fabrics under combined temperature, humidity, irradiation, chemical media. The damage mechanism can be divided into two processes: physical damage arises from thermal stress and moisture intrusion, and chemical degradation caused by photo–oxygen–water coupling.

Due to the different thermal expansion coefficients between the polyurethane coating and the polyester/metal fiber substrate, periodic thermal–mechanical stress reduces the interfacial bonding strength [56]. SEM reveals pores and cracks, significantly reduced adhesion and thinning, and exposed fibers. These defects facilitate oxygen, water, and Cl^−^ penetration [9]. At the same time, elevated temperatures accelerate degradation reaction kinetics and hasten fabric aging. Water permeating coating pores induces swelling and weakens coating–matrix interfacial cohesion [18]. It disrupts the arrangement of fibers, exacerbates coating damage, and accelerates substrate aging [56].

Irradiation cleaves C–N and C–O bonds, generating amino, alkyl and carbamoyl, alkoxy radicals [11]. Through a series of reactions, these generate –NH_2_ and –CHO, and release CO_2_, culminating in the photodegradation of –NHCOO– [32,57]. Hydrolysis also breaks the molecular chains of polyurethane coating. Water penetration triggers hydrolysis of –NHCOO–, producing –NH_2_ and –OH [16]. Simultaneously, hydrolysis of the polyether soft segment C–O–C generates –OH. Moreover, ether bonds are oxidized to form –COOH [16,51]. Corrosive media with permeability, such as Cl^−^, quickly pass through the pores and penetrate into the fabric, thereby accelerating damage [55]. Exposed metals/metal compounds further degrade fibers and weaken coating–fiber adhesion.

In BN, extreme humidity and heat combined with intense irradiation synergistically drive continuous uniform degradation of polyurethane coatings. Coupled photothermal oxidative chain scission and hydrolysis cause severe delamination. This manifests as drastic deterioration with gloss at 0.2, an 80% decrease; color difference at 6.93; and severe deterioration of mechanical properties with a retention rate of only 31%. In XM, the penetration of Cl^−^ ions in salt spray induces electrochemical corrosion, which accelerates surface chalking and erosion of the coating, thus causing a sharp decline in optical and mechanical performance. Gloss is 0.4, a decrease of 60%; color difference is 6.34; and the mechanical property retention rate is 48%. In JN, thermomechanical fatigue from thermal cycling propagates interfacial microcracks, yet overall structural integrity remains comparatively preserved. After aging, measurements show an 80% gloss decrease to 0.2, a color difference ΔE* of 5.89, and a 50% mechanical retention. Multifactorial degradation ultimately destabilizes coating/substrate interfaces, causing discoloration, fiber damage, and mechanical decline.

## 4. Conclusions

The natural aging behavior of polyurethane-coated fabrics was evaluated through 24-month exposure tests under multi-environmental factors in BN, XM, and JN over 24 months, and the following conclusions were drawn:

(1) The combined effect of these chemical and physical mechanisms resulted in visible chalking, thinning, fiber exposure, and fiber damage after 24 months of exposure. Consequently, the gloss retention rate was no greater than 40%, the color difference exceeded 5.8, and mechanical properties were reduced by more than 50%. Region BN exhibited the most severe degradation, followed by XM and JN.
(2) In BN, high solar irradiation served as the primary accelerator, intensifying oxidation and hydrolysis. In XM, high salinity reduced coating adhesion and damaged the substrate, exacerbating degradation in coastal conditions. In JN, cyclic thermal stress resulting from temperature variations weakened interfacial bonding.
(3) The degradation is primarily induced by chemical processes including hydrolysis and photodegradation of polyurethane, oxidation, and hydrolysis of ether bonds. These are accompanied by physical damage mechanisms such as swelling and thermal stress.

## Figures and Tables

**Table 1:** Climate conditions during natural aging tests in different regions.

Location	$\bar{T}$(°C)	T_max_ (°C)	T_min_ (°C)	RH (%)	Rf (mm)	U_max_ (m/s)	SD (h)	I_s_ (MJ/m^2^)
BN	22.6	40.8	9.7	73	1327.8	4.50	2235	6396.90
XM	22.1	36.7	4.8	79	1279.7	9.55	2233	5558.48
JN	15.4	39.0	−17.7	52	660.3	10.14	2568	5871.58

$\bar{T}$ is the average temperature. T_max_ is the maximum temperature. T_min_ is the minimum temperature. RH is the relative humidity. Rf is the average annual rainfall. U_max_ is the maximum wind speed. SD is the annual sunshine duration. I_s_ is the annual solar irradiance. The data is sourced from the European Centre for Medium-Range Weather Forecasts and climate bulletins of local governments.

**Table 2:** Degree and grade of discoloration (GB/T 1766-2008).

Degree	ΔE*	Grade
0	≤1.5	No color change
1	1.6~3.0	Very slight color change
2	3.1~6.0	Slight color change
3	6.1~9.0	Distinct color change
4	9.1~12.0	Considerable color change
5	>12.0	Severe color change

## Data Availability

The original contributions presented in this study are included in the article; further inquiries can be directed to the corresponding author.

## Abbreviations

The following abbreviations are used in this manuscript:

UV	Ultraviolet rays
BN	Xishuangbanna, China
XM	Xiamen, China
JN	Jinan, China
$\bar{T}$	Average temperature
T_max_	Maximum temperature
T_min_	Minimum temperature
RH	Relative humidity
Rf	Average annual rainfall
SD	Annual sunshine duration
U_max_	Maximum wind speed
I_s_	Annual solar irradiance
ΔE	Color difference
ATR-FTIR	Attenuated total reflection Fourier-transform infrared
XPS	X-ray photoelectron spectroscopy
SEI	Secondary electron images
BSE	Backscattered electron images
